# Adopted Children

**Published:** 1920-03-27

**Authors:** 


### Adopted Children.

The great increase during recent years in the practice of child adoption has led to the consideration of proper legislation to safeguard the children. It has become fashionable to adopt a child, and a fashion of this kind—followed as a fashion—is liable to abuse. Some Boards of Guardians have also had their eyes on it with the object of preventing foster-parents from taking children for the sole purpose of making economic use of them. It certainly needs watching, for such children ought not to be allowed to be exploited, or, as is said to be occasionally so, transferred from one house to another, regardless of their feelings and interests, just because the foster parents have either tired of their effort, or do not find it suits their purpose to continue to keep the children.

A deputation from the National Council of Women waited on the Home Secretary last week, to urge him to introduce legislation legalising the adoption of children. In introducing the deputation, Sir J. G. Butcher, M.P., said the necessity for legislation had been emphasised by the conditions brought about by the war. Adoption agreements, he said, should be regularised and legalised so that a person who adopted a child should be legally bound to carry out all responsibilities and obligations. He hoped the Home Secretary would think it desirable to appoint a committee of experts to report on the question.

Mrs. Ogilvie Gordon, President of the National Council of Women, said the deputation represented the whole of the thinking women of the country, who felt that the matter should be attended to. Many other countries, she said, were far in advance of England on that question.

Mrs. Edwin Gray said promiscuous adoption was going on which was fraught with danger for the child. The time had come when adoption should be legally sanctioned, and some formalities should be necessary before a Court before anyone adopted a child. Unscrupulous lawyers were now giving deeds for a guinea, and were telling the people that the child was legally their own. She did not like the idea of a child being handed out on probation like a cat or a dog.

Miss Rosamund Smith said that adoption was now made a cloak for baby farming, and that an adopted child when it became old was used to help the family budget.

Mr. R. Newton Crane said that England was the only civilised country in the world in which adoption was not legalised.

The Home Secretary, in reply, said that the subject had been raised a good many times, and was not entirely non-contentious. A good many people looked upon children as a legitimate investment for their old age. He could make no promise with regard to legislation, but he would consider Sir John Butcher's suggestion of appointing a Committee to investigate.

